# Meta-analysis and network meta-analysis of time-to-event outcomes with non-proportional hazards: a Bayesian time-varying hazard ratio approach

**DOI:** 10.1186/s12874-026-02963-5

**Published:** 2026-08-04

**Authors:** Rhiannon K. Owen, Keith R. Abrams

**Affiliations:** 1 Swansea University Medical School, Swansea University, Singleton Park, SA2 8PP Swansea, Wales UK; 2 Department of Statistics, University of Warwick, Mathematical Sciences Building, CV4 7AL Coventry, England UK; 3 Warwick Medical School (WMS), University of Warwick, Gibbet Hill, CV4 7AL Coventry, England UK; 4 Centre for Health Economics, University of York, Heslington, YO10 5DD York, England UK

**Keywords:** Meta-analysis, Network meta-analysis, Bayesian methods, Non-proportional hazards, Time-varying hazard ratio

## Abstract

**Background:**

Meta-analyses of time-to-event (TTE) outcomes, particularly in Health Technology Assessment (HTA), commonly use a hazard ratio (HR) scale. However, non-proportional hazards in included trials create difficulties. Existing methods are either too complex or not easily incorporated into economic decision models for cost-effectiveness assessment.

An alternative approach assumes a treatment-log(time) interaction within a Cox proportional hazards model, allowing the log HR to vary linearly with log(time). A bivariate meta-analysis of the resulting treatment and interaction coefficients then yields an overall time-varying HR (TVHR) with appropriate uncertainty.

**Methods:**

The TVHR approach was applied to a meta-analysis of 20 trials (4,069 patients) comparing chemotherapy to Standard of Care (SoC) for advanced recurrent gastric cancer, with Progression-Free Survival (PFS) as an outcome (median follow-up 1.2 years). It was also applied to a network meta-analysis (NMA) of 13 treatments across 13 Randomised Controlled Trials (RCTs) in previously untreated advanced BRAF-mutated melanoma (3,913 deaths in 6,378 participants) evaluating Overall Survival (OS). Both applications were compared against standard Bayesian meta-analysis assuming proportional hazards.

**Results:**

Five trials in the gastric cancer meta-analysis showed non-proportional hazards for PFS. A standard Bayesian random-effects meta-analysis yielded a pooled HR of 0.78 (95% CrI: 0.70–0.86). The TVHR model produced a pooled interaction effect of 0.09 (95% CrI: -0.005-0.193), with HRs ranging from 0.83 (95% CrI: 0.75-0.91) at 0.5 years to 0.99 (95% CrI: 0.79-1.23) at 3.5 years. Three studies in the melanoma NMA showed non-proportional hazards for OS. Using the TVHR model, nivolumab plus ipilimumab demonstrated consistent superiority from month 7, with improving HRs from 0.37 (95% CrI: 0.26-0.51) at one year to 0.24 (95% CrI: 0.12-0.45) at five years (mean rank 1.18, 95% CrI: 1-2). Dabrafenib- and vemurafenib-based regimens showed evidence of waning treatment effects, potentially becoming inferior to dacarbazine (HR>1) at five years.

**Conclusions:**

The TVHR approach offers a simple, intuitive solution for meta-analysis of TTE outcomes when proportional hazards do not hold, and are readily incorporated into economic decision models. Extensions to a fully Bayesian one-stage model, and spline-based interaction effects are also feasible.

**Supplementary Information:**

The online version contains supplementary material available at https://doi.org/10.1186/s12874-026-02963-5.

## Introduction

Often when conducting meta-analyses (MA) of time-to-event (TTE) results, especially in Health Technology Assessment (HTA), a hazard ratio (HR) scale is used due to the ease with which a treatment effect can be incorporated into a cost-effectiveness decision model [1, 2]. However, issues arise when there is evidence of non-proportional hazards in some of the trials/studies included. In a network meta-analysis setting, departures from proportional hazards introduce additional complexity, as the assumption of consistency between direct and indirect treatment comparisons may be violated when HRs vary over time [3].

When Individual Participant Data (IPD) are available, or have been re-created from published Kaplan-Meier estimates [4], a number of methods have been advocated, including; parametric models [5, 6], flexible parametric models [7], piecewise exponential models [8], fractional polynomial models [3], flexible M-splines [9], and Restricted Mean Survival Time (RMST) models [10]. However, their use has been limited by either their complexity and/or the ease with which their results can be incorporated into an economic decision model in order to assess cost-effectiveness. A further contributing factor is the current absence of consensus-based, impartial guidance on when a given non-proportional hazards method should be preferred. We acknowledge that the TVHR approach proposed here adds to an already large set of available methods, and we do not claim it as a universally preferred solution; rather, we present it as a pragmatic option that may be preferred in settings that value transparency, ease of implementation using standard Cox model outputs, and direct compatibility with hazard-ratio-based economic decision models, while acknowledging that other, more flexible methods may be preferable where these properties are less important than capturing more complex hazard trajectories.

In a HTA setting, a HR is usually applied to a target baseline population (with or without treatment waning) [1, 2]. Whilst some of the existing methods enable a HR to be estimated others do not. Treatment effect waning scenarios are also more easily incorporated and interpreted on a HR scale, as it is a relative measure [11].

In this paper we present a simple Time-Varying Hazard Ratio (TVHR) approach - which assumes that the HR in each study in a meta-analysis can change linearly with respect to log(time) and the resulting coefficients from each study are then synthesised using a bivariate meta-analysis to yield an overall relationship between the log HR and log(time) [12]. A key feature of the proposed framework is that the standard proportional hazards meta-analysis arises as a special case when the coefficient on log(time) is zero.

The rest of the paper is organised as follows; Motivating examples section introduces two motivating examples - one a pairwise meta-analysis of treatments for advanced gastric cancer, and the second a network meta-analysis of 13 treatments for advanced BRAF-mutated Melanoma - in both cases there was evidence of non-proportional hazards in some of the studies included. Methods section describes the methods which can be used to undertake a pairwise or network meta-analysis when either proportional hazards is assumed to hold or when the assumption is relaxed and a TVHR approach adopted, whilst Results section presents the results of applying a TVHR approach to the two motivating examples. Finally, Discussion and Conclusion sections discuss some of the advantages and limitations of a TVHR approach and present conclusions.

## Motivating examples

### Pairwise meta-analysis - advanced gastric cancer

**Fig. 1:**
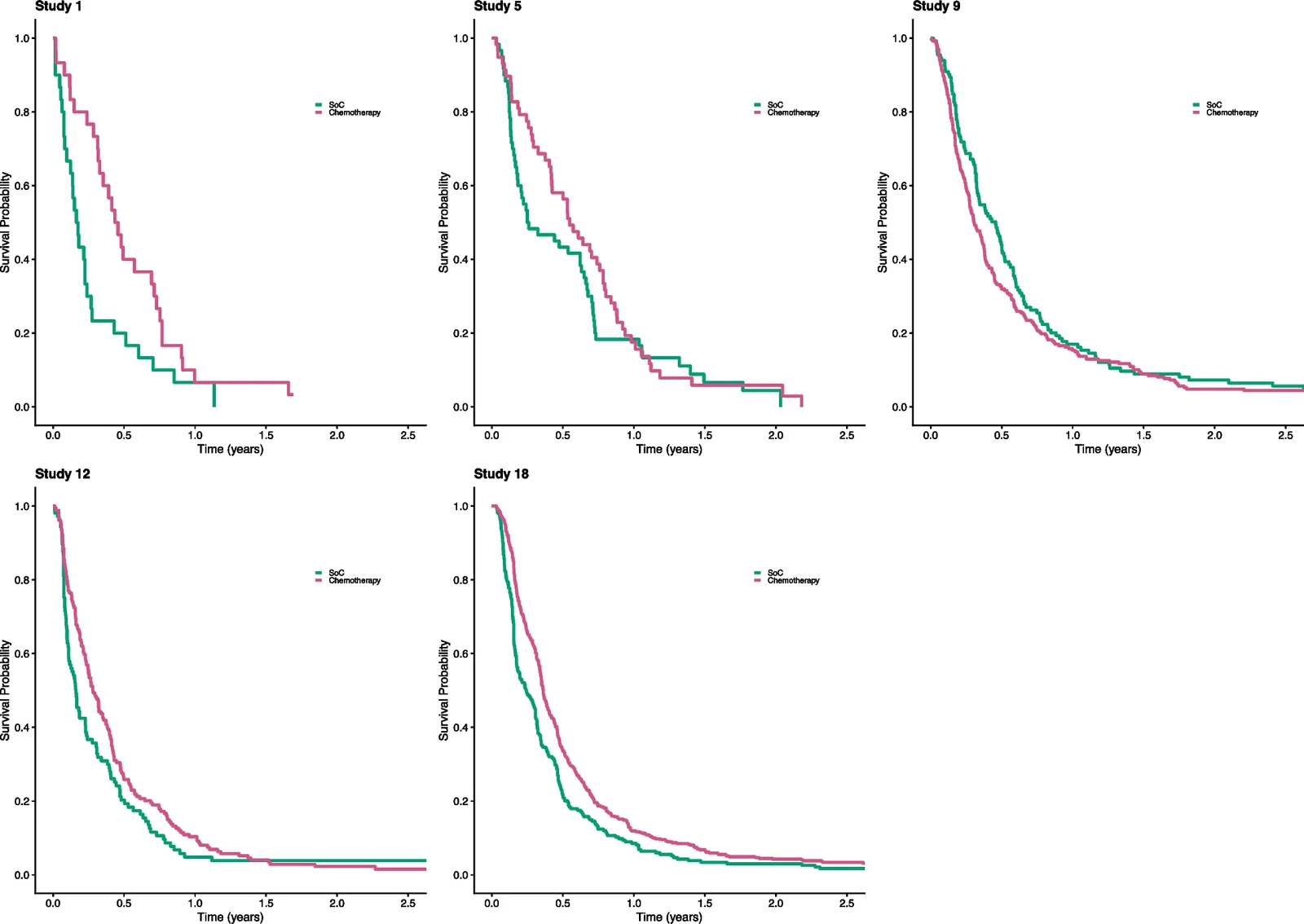
Gastric cancer meta-analysis - Kaplan-Meier estimates for studies with non-proportional hazards

An IPD meta-analysis of 20 trials, ranging in size from 58 patients to 704 patients, involving 4,069 patients, comparing chemotherapy to Standard of Care (SoC) for advanced recurrent gastric cancer was undertaken by the Global Advanced/Adjuvant Stomach Tumour Research International Collaboration (GASTRIC) Group [13] & [14]. The primary outcome was Progression-Free Survival (PFS) with a median follow-up of 1.2 years and up to a maximum of 7.2 years during which 3,820 events were observed. Of the 20 trials in the meta-analysis 5 (Study 1, P=0.04; Study 5, P=0.09; Study 9, P=0.04; Study 12, P=0.02 & Study 18, P=0.0004) displayed evidence of non-proportional hazards for PFS as can also be seen from the Kaplan-Meier and Schoenfeld residual plots in Figs. 1 & A.1 (see Additional File A). However, it should be noted that formal testing for departures from the assumption of proportional hazards has been shown to have low statistical power [15].

### Network meta-analysis - advanced BRAF-mutated melanoma

**Fig. 2:**
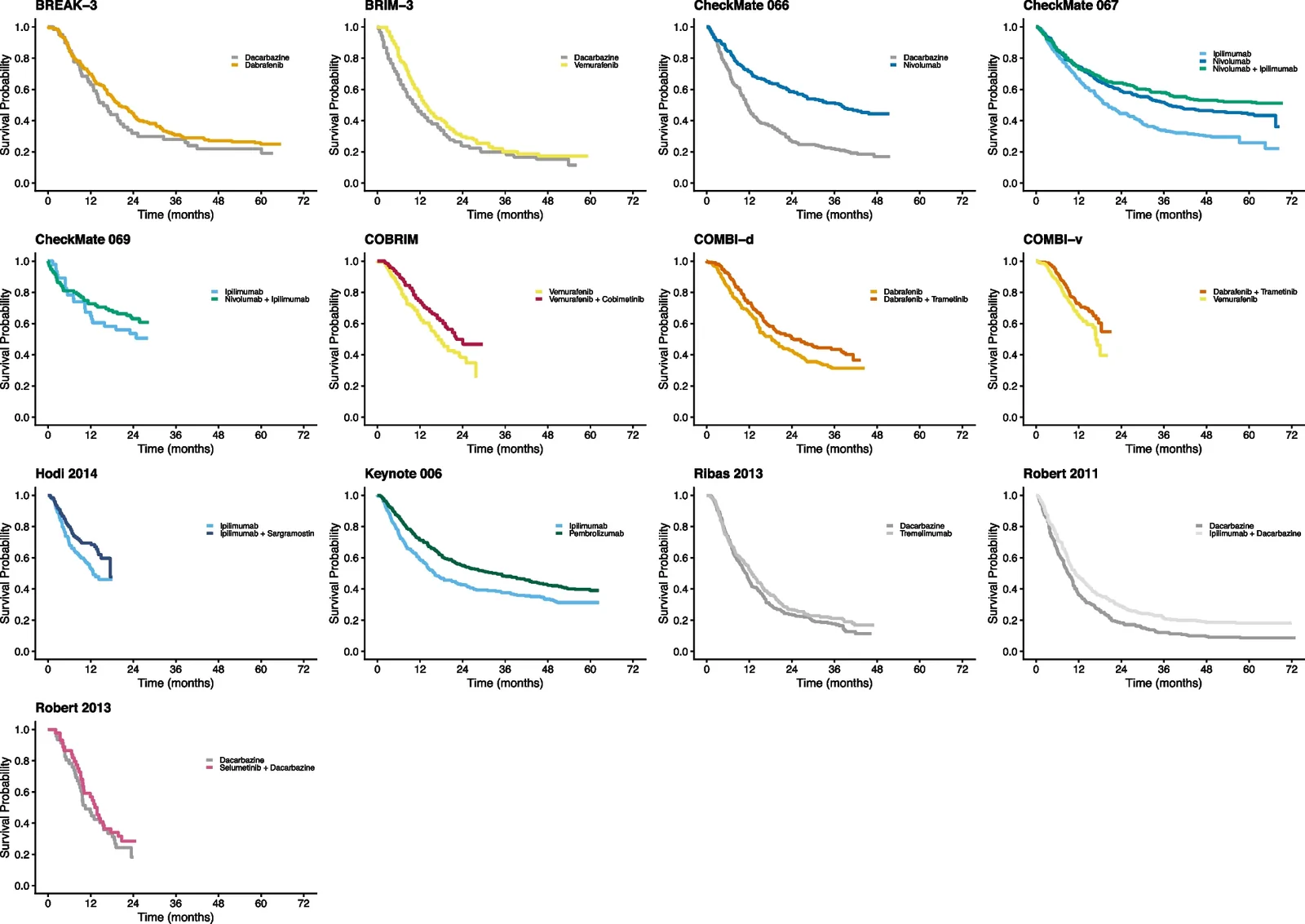
Advanced BRAF-mutated melanoma NMA - Kaplan-Meier estimates

A network meta-analysis in previously untreated advanced BRAF-mutated melanoma as discussed in [16] with full IPD extracted and reported in [17] was used as a motivating example. The primary outcome was overall survival with median follow-up ranging from 1 year to 5.3 years. The network included 13 RCTs and 13 treatments including dabrafenib (DB), dacarbazine (DTIC), ipilimumab (IPI), tremelimumab (TRL), nivolumab (NIV), pembrolizumab (PEM), and vemurafenib (VM), as single treatments, and dabrafenib + trametinib (DB + TR), ipilimumab + dacarbazine (IPI + DTIC), ipilimumab + sargramostin (IPI + SRG), nivolumab + ipilimumab (NIV + IPI), selumetinib + dacarbazine (SEL + DTIC) and vemurafenib + cobimetinib (VM + COB), as combination treatments (Fig. B.1, see Additional File B). There were 12 two-arm trials, and 1 three-arm trial included in the network with the majority of direct treatment comparisons informed by single studies. In total, 6,378 participants were included in the network, with 3,913 mortality events. The sample size of included studies ranged from 45 to 556 participants. Figure 2 illustrates the Kaplan-Meier plots for all 13 studies. Inspection of Schoenfeld residual plots suggested possible time-varying effects across several studies (Fig. B.2, see Additional File B). However, evidence from global tests of proportional hazards indicated statistically significant deviations at the 5% level in three studies (BRIM-3, CheckMate-067, and Keynote 006).

## Methods

### Cox proportional hazards models & time-varying hazard ratio models

The standard Cox proportional hazards regression model is given by (1) in which $x_i$ represents a binary treatment indicator and *β * the log-hazard ratio,

1 $$\begin{aligned} \lambda (t) = \lambda _0(t) \exp (\beta x_i) \end{aligned}$$

and $\lambda _0(t)$ is an unspecified baseline hazard function with *β * estimated via a partial likelihood [18]. The standard Cox PH model in (1) can be extended to allow for a time-varying (log-)hazard ratio by including an interaction term between the treatment indicator and log(time) [12],

2 $$\begin{aligned} \lambda (t) = \lambda _0(t) \exp (\beta _1 x_i + \beta _2 x_i \log (t)) \end{aligned}$$

The hazard ratio at time *t* is then given by $\exp (\hat{\beta _1}+\hat{\beta _2} \log (t))$. Models such as (2) can be easily fit in R [19] using the following code [12];

**Figure:**
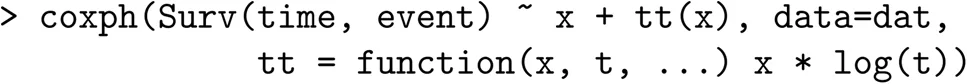

where the variables time, event and x present the time to an event, event indicator and binary covariate respectively in the data frame, dat.

### Pairwise meta-analysis model

#### Cox proportional hazards models

For study $i = 1,\dots ,n_s$ with two arms, let $y_i$ denote the estimated log hazard ratio for the treatment arm compared to control, obtained from Cox proportional hazards models such as (1). We assume

3 $$\begin{aligned} y_i \sim \textrm{N}\!\left( \delta _i,\, S_i\right) ,\qquad i=1,\dots ,n_s , \end{aligned}$$

where $S_i$ is the estimated within-study variance which is assumed to be known. The true relative treatment effects, $\delta _i$, are modelled using a standard pairwise meta-analysis random-effects formulation;

4 $$\begin{aligned} \delta _i \sim \textrm{N}\!\left( d\; , \; \tau ^2 \right) , \end{aligned}$$

Adopting a Bayesian formulation, the overall pooled log hazard ratio, *d*, is often assumed to have a relatively vague prior distribution such that, $d \sim \textrm{N}(0,1000)$, whilst the between-study standard deviation, *τ *, can be assumed to have a $\textrm{Unif}(0,1)$ prior distribution. Such a $\textrm{Unif}(0,1)$ prior distribution implies that the HRs of two random studies could differ by a factor of 2.97 and the range of minimum to maximum HRs could differ by a factor of 50 [20]. Other prior distributions for *τ * could include informative prior distributions based on meta-research [21]. Estimation of *d* and *τ * is most usually undertaken via Markov Chain Monte Carlo (MCMC) [22].

#### Time-varying hazard ratio models

For each study $i = 1,\dots ,n_s$, let $\textbf{y}_i$ denote the bivariate vector of estimated coefficients from a model such as (2) with $\textbf{y}_i = (y_{i1},y_{i2})$ where $y_{i1}$ is the log hazard ratio for treatment, and $y_{i2}$ is the treatment-log(time) interaction. Further, let the within-study covariance matrix be $\textbf{S}_i$ and which is assumed to be known. Assuming multivariate Normality (MVN) on the log hazard ratio scale the likelihood is thus

5 $$\begin{aligned} \textbf{y}_i \sim \text {MVN}\!\left( \boldsymbol{\delta }_{i},\; \textbf{S}_i \right) , \qquad i=1,\dots ,n_s . \end{aligned}$$

where $\boldsymbol{\delta }_{i} = (\delta _{i1},\delta _{i2})$ represents the vector of true relative effects for treatment log hazard ratio and the treatment-log(time) interaction.

Further $\boldsymbol{\delta }_{i}$ is assumed to follow a bivariate random-effects model:

6 $$\begin{aligned} \boldsymbol{\delta }_{i} \sim \text {MVN}\!\left( \textbf{d},\; \boldsymbol{\Sigma } \right) \end{aligned}$$

in which $d_1$ and $d_2$, the pooled treatment log hazard ratio and treatment-log(time) interaction respectively, are assumed to have independent *N*(0, 1000) vague prior distributions and the *2 × 2* between-study covariance matrix, $\boldsymbol{\Sigma }$, is defined such that

7 $$\begin{aligned} \boldsymbol{\Sigma } = \left( \begin{array}{cc} \tau _1^2 & \rho \, \tau _1 \tau _2 \\ \rho \, \tau _1 \tau _2 & \tau _2^2 \end{array}\right) , \end{aligned}$$

and is estimated using a spherical parameterisation under an assumption of homogeneous between-study variances and correlation [23] such that

8 $$\begin{aligned} \tau _m&\sim \text {Unif}(0,1), \qquad m=1,2, \end{aligned}$$

9 $$\begin{aligned} \theta&\sim \text {Unif}(0,\pi ), \qquad \rho = \cos (\theta ). \end{aligned}$$

In order to explore sensitivity to the prior distribution specification for the treatment-log(time) interaction parameter, $\tau _2$, in the between-study covariance, $\boldsymbol{\Sigma }$, a Half-Normal(0,0.5) prior distribution can also used [24].

### Network meta-analysis

#### Cox proportional hazards models

For study $i = 1,\dots ,n_s$ with $n_a(i)$ arms, let $y_{i,(k-1)}$ denote the estimated log hazard ratio for arm $k=2,\dots ,n_a(i)$ relative to the study-specific reference arm (*k=1*), obtained from Cox proportional hazards models. We assume

10 $$\begin{aligned} y_{i,(k-1)} \sim \textrm{N}\!\left( \delta _{i,t[i,k]}, \; S_{i,(k-1)}\right) , \quad k=2,\dots ,n_a(i), \end{aligned}$$

where $S_{i,(k-1)}$ is the known within-study variance. The true relative treatment effects are modelled using a conditional formulation to account for correlations induced by multi-arm trials:

11 $$\begin{aligned} \delta _{i,t[i,k]} \sim \textrm{N}\left( d_{t[i,k]} - d_{t[i,1]} + \frac{1}{k-1} \sum \limits _{a=1}^{k-1} w_{i,a}, \; \frac{2(k-1)}{k}\tau ^2 \right) , \quad k=2,\dots ,n_a(i), \end{aligned}$$

where the study-specific random effects are defined as

12 $$\begin{aligned} w_{i,k}&= \delta _{i,t[i,k]} - \left( d_{t[i,k]} - d_{t[i,1]}\right) , \quad k=1,\dots ,n_a(i), \nonumber \\ \delta _{i,t[i,1]}&= 0. \end{aligned}$$

The basic parameters $d_t$ represent pooled log hazard ratios relative to treatment 1, with prior distributions

13 $$\begin{aligned} d_t&\sim \textrm{N}(0,1000), \quad t=2,\dots ,n_t, \nonumber \\ d_1&= 0. \end{aligned}$$

Between-study heterogeneity is captured by a common variance parameter, *τ *, such that

14 $$\begin{aligned} \tau \sim \textrm{Unif}(0,1). \end{aligned}$$

#### Time-varying hazard ratio models

Let $\textbf{y}_{i,(k-1)}$ be the estimated *m*-vector of coefficients for arm $k = 2,...,n_a(i)$ versus the study-specific reference arm, *k=1* such that *m=1* represents the log hazard ratio for the treatment effect, and *m=2* the interaction of treatment and log-time, obtained from Cox proportional hazards models for study $i = 1,2,...,n_s$. Let $\textbf{y}_{i,(k-1)}$ follow a multivariate normal distribution, with known contrast-specific within-study covariance, $\textbf{S}_{i,(k-1)}$, obtained from Cox proportional hazards models such that:

15 $$\begin{aligned} \textbf{y}_{i,(k-1)} \sim \text {MVN}\!\left( \boldsymbol{\delta }_{i,t[i,k]}, \textbf{S}_{i,(k-1)}\right) , \quad k=2,\dots ,n_a(i). \end{aligned}$$

The true coefficients for the relative treatment effect, $\boldsymbol{\delta }_{i,t[i,k]}$, for treatment *t*[*i*, *k*], in study *i* arm *k* is given by

16 $$\begin{aligned} \boldsymbol{\delta }_{i,t[i,k]} \sim \text {MVN} \left( \textbf{d}_{t[i,k]} - \textbf{d}_{t[i,1]} + \frac{1}{(k-1)} \sum \limits _{a=1}^{(k-1)} \textbf{w}_{i,a}, \; \frac{2(k-1)}{k}\boldsymbol{\Sigma }\right) , \quad k=2,\dots ,n_a(i) \end{aligned}$$

The conditional covariance for arm *k* is given by $\frac{2(k-1)}{k}\boldsymbol{\Sigma }$. For *k=2* this reduces to *Σ *, which is the marginal of the first contrast. The conditional mean is calculated using the average of the previous ${\textbf {w}}_{i,a}$ to apply an appropriate multi-arm correction to the covariance structure, where

17 $$\begin{aligned} \textbf{w}_{i,k}&= \boldsymbol{\delta }_{i,t[i,k]} - (\textbf{d}_{t[i,k]} - \textbf{d}_{t[i,1]}),\quad k=1,\dots ,n_a(i) \end{aligned}$$

18 $$\begin{aligned} \boldsymbol{\delta }_{i,t[i,1]}&= \textbf{0}, \end{aligned}$$

The *m*-vector of pooled treatment effects for treatment, *t*[*i*, *k*], in arm *k* of study *i*, relative to the study-specific reference arm, *t*[*i*, 1], is indicated by $\textbf{d}_{t[i,k]}$, with non-informative prior distributions assigned to the basic parameters for each coefficient *m* such that

19 $$\begin{aligned} d_{t,m}&\sim \text {N}(0, 1000), \quad \text {for} \quad t=2,\ldots , n_{t},\nonumber \\ d_{1,m}&= 0 \quad m=1,2, \end{aligned}$$

The *m × m* between-study covariance, $\boldsymbol{\Sigma }$, is estimated using a spherical parameterisation under an assumption of homogeneous between-study variances [25] and correlations across treatment contrasts [26] such that

20 $$\begin{aligned} \boldsymbol{\Sigma }&= \left( \begin{array}{cc} \tau _1^2 & \rho \, \tau _1 \tau _2 \\ \rho \, \tau _1 \tau _2 & \tau _2^2 \end{array}\right) , \end{aligned}$$

21 $$\begin{aligned}&\tau _m \sim \text {Unif}(0,1), \quad m=1,2, \end{aligned}$$

22 $$\begin{aligned} \theta&\sim \text {Unif}(0,\pi ), \quad \rho = \cos (\theta ). \end{aligned}$$

### Model fitting

Models were fitted in R [19] and JAGS [27] using rjags [28]. All models were run for 50,000 iterations of burn-in followed by 50,000 posterior samples across three chains, with a thinning factor of 5, yielding a retained sample of 10,000 draws per chain (30,000 draws across chains). The convergence of MCMC chains was assessed for basic parameters using the Gelman-Rubin potential scale reduction factor (PSRF), $\hat{R}$, calculated for each monitored parameter in three independent chains [29]. The PSRF compares between-chain variance to within-chain variance, with values close to 1.00 indicating that the chains have converged to a common stationary distribution. Values of $\hat{R}<$ 1.01 were used as the threshold for convergence [30]. Effective sample size (ESS) was computed for all monitored parameters using the effectiveSize function in the coda package [31], with a minimum ESS of 1000 per parameter considered sufficient for reliable posterior inference with regards to means and credible intervals. Convergence of MCMC chains was visually assessed through autocorrelation and trace plots [31]. All analyses were undertaken using an annual time scale in order to ensure adequate mixing of MCMC chains. All code for fixed and random effects models is provided at: https://github.com/r-k-owen/NMA-TVHR.

## Results

### Pairwise meta-analysis - advanced gastric cancer

Applying a Bayesian 2-stage meta-analysis using (3) & (4) to the advanced gastric cancer meta-analysis produces an overall posterior HR of 0.78 with 95% CrI (0.70 to 0.86) (Table 1). These results were similar to both a frequentist 2-stage and 1-stage meta-analysis (see Additional File A, Fig. A.2). The very slightly wider interval obtained using a Bayesian approach being due to allowing for the uncertainty in the estimation of the between-study variance, $\tau ^2$.

**Table 1:** Gastric cancer meta-analysis - pooled constant & time-varying hazard ratios (HR) and associated 95% credible intervals (CrI) for chemotherapy versus standard of care

Time (Years)	HR	95% CrI
Constant	0.78	0.70 to 0.86
0.5	0.83	0.75 to 0.91
1.0	0.88	0.78 to 0.99
1.5	0.82	0.79 to 1.06
2.0	0.94	0.79 to 1.11
2.5	0.96	0.79 to 1.15
3.0	0.98	0.79 to 1.19
3.5	0.99	0.79 to 1.23

**Fig. 3:**
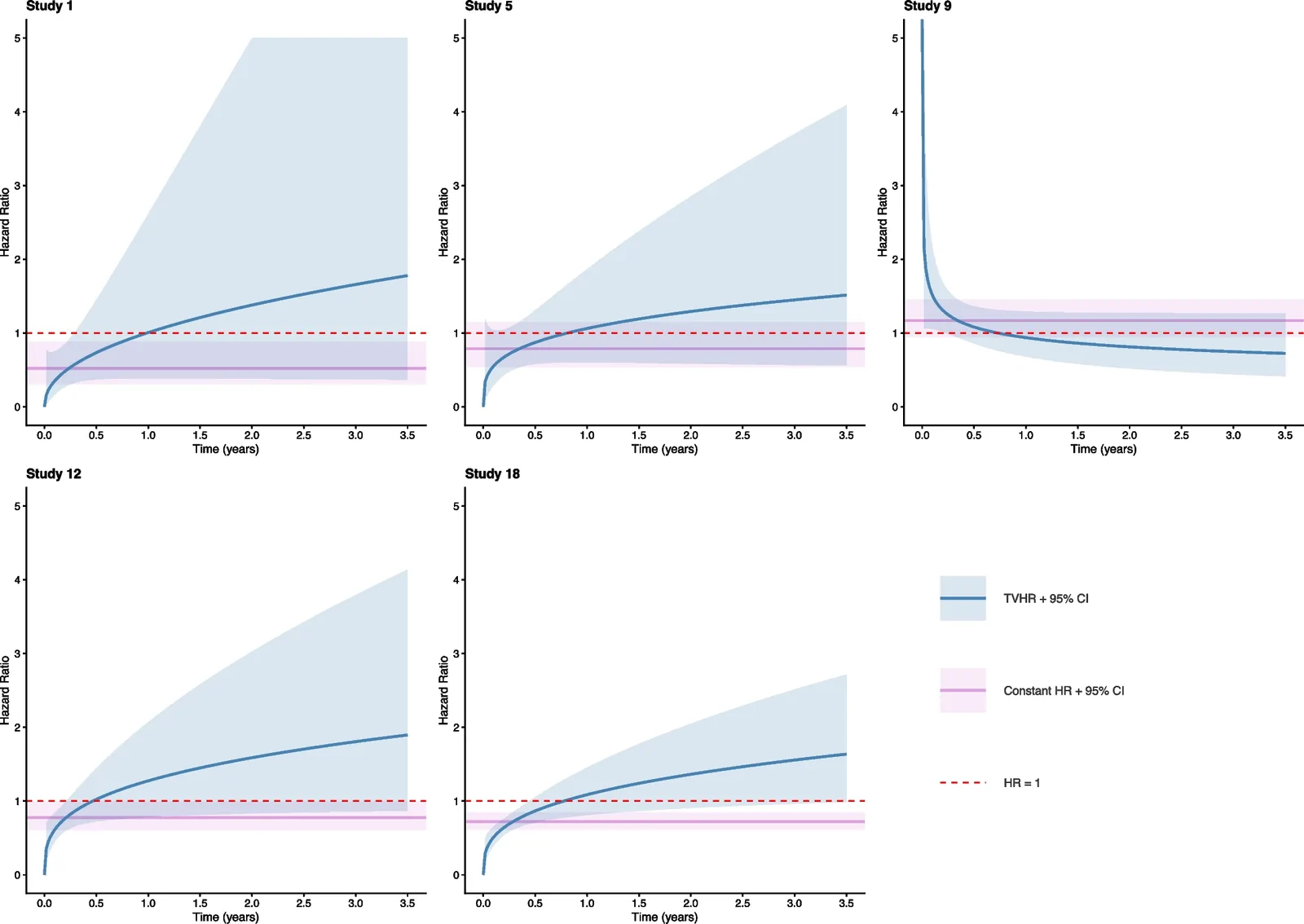
Gastric cancer meta-analysis - study-specific time-varying (TVHR) and constant hazard ratios, and associated 95% confidence intervals (CI)

Figure 3 displays the results of fitting a TVHR model (2) to each of the 5 studies which display some evidence of non-proportional hazards in Figs. 1  & A.1 (see Additional File A) compared to a standard Cox model (1) and it can be seen that there are substantial discrepancies in terms of the resulting HRs at both earlier and later time points. Of note, Study 9 exhibits a treatment-log(time) interaction in the opposite direction to the other four studies with evidence of non-proportional hazards: rather than an increasing HR over time (waning chemotherapy benefit), the study-specific TVHR for Study 9 decreases over time, implying an improving relative benefit of chemotherapy with longer follow-up (Fig. 3). Because Study 9 is one of only five studies contributing to the interaction term in the bivariate synthesis, and because the between-study correlation and heterogeneity parameters are estimated from this same small set of studies, this discordant study could disproportionately influence both the pooled interaction estimate and its uncertainty. We do not consider this sufficient justification to exclude Study 9 from the primary analysis, since no methodological reason for the discordance is apparent (e.g. in trial design, population, or follow-up), but we flag this as a study that decision-makers and reviewers of any subsequent cost-effectiveness analysis may wish to examine, and we recommend a leave-one-out sensitivity analysis excluding Study 9 be reported alongside the primary TVHR results in applied use of this method.

**Fig. 4:**
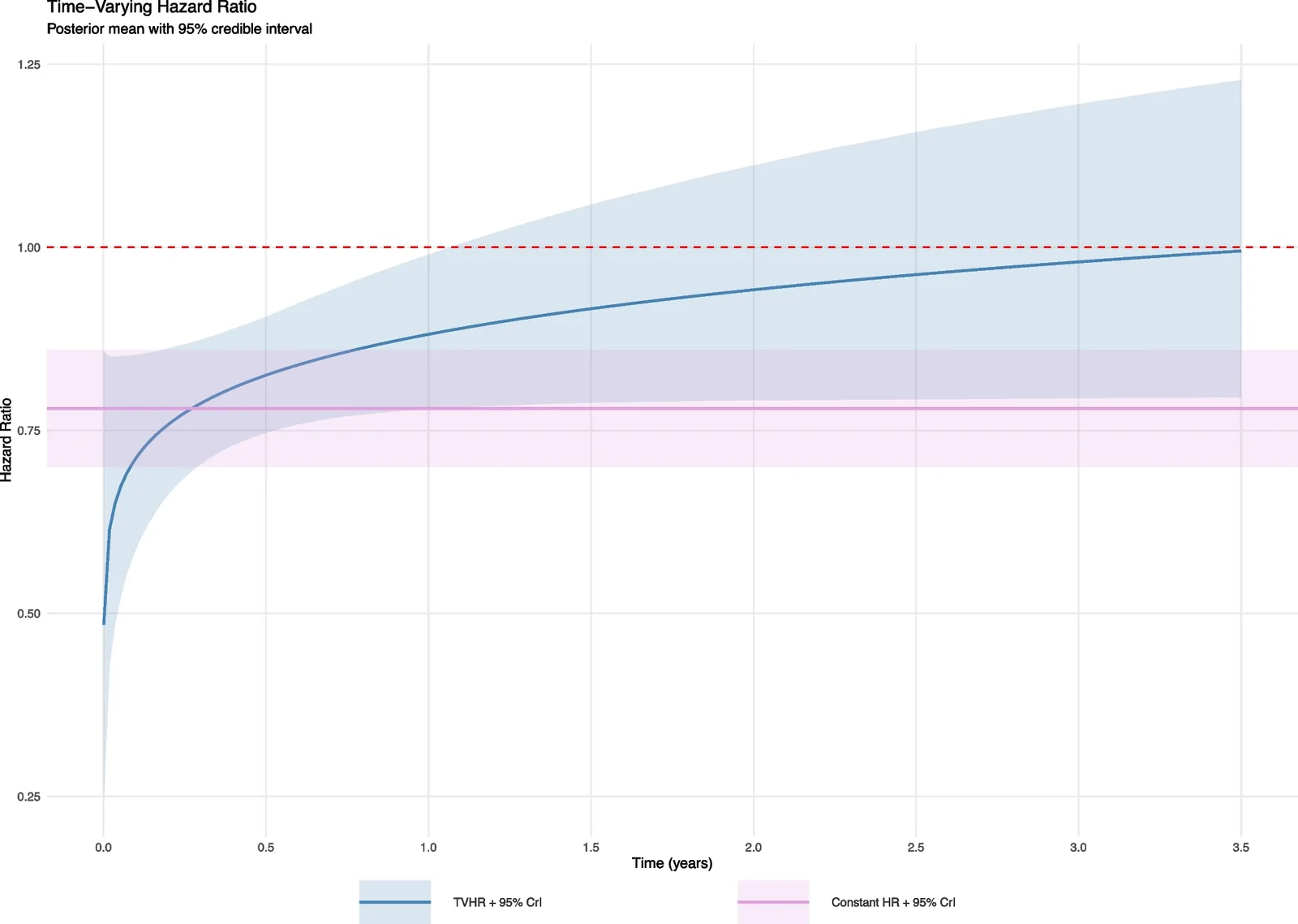
Gastric cancer meta-analysis - pooled time-varying (TVHR) and constant hazard ratio, and associated 95% credible intervals (CrI)

Figure 4 and Table 1 show the results of fitting the Bayesian 2-stage TVHR bivariate meta-analysis model (5)-(7) in comparison to a Bayesian 2-stage constant HR meta-analysis model (3) & (4) in terms of the pooled HRs and 95% CrIs. All values of $\hat{R}<$ 1.01, and ESS >1000 per parameter, suggesting that the MCMC chains have converged to a common stationary distribution with reliable posterior inference. The 2-stage TVHR bivariate model produces a posterior mean for the treatment-log(time) interaction parameter of 0.09 with 95% CrI (-0.005 to 0.193) and a posterior probability that the interaction effect is positive of 0.97, providing supportive evidence that allowing for a TVHR is warranted. It can be seen that, as with the study-specific results (Fig. 3), there are substantial discrepancies between the two approaches at both earlier and later time points. In particular, the upper limit of the 95% CrI for the TVHR approach is above 1 (no treatment difference) at time points beyond 1 year and by 3 years the posterior mean for the HR is also very close to 1 indicating no benefit of chemotherapy over SoC, whilst the standard 2-stage, approach assuming a constant HR, confers a 22% relative risk reduction (of progression or death) to Chemotherapy over SoC at *all* time points with a 95% CrI from a 14% reduction to a 30% reduction. The sensitivity analysis in Table A.1 (see Additional File A) showed that the results are invariant to specification of a Half-Normal(0,0.5) prior distribution for $\tau _2$.

### Network meta-analysis - advanced BRAF-mutated melanoma

**Fig. 5:**
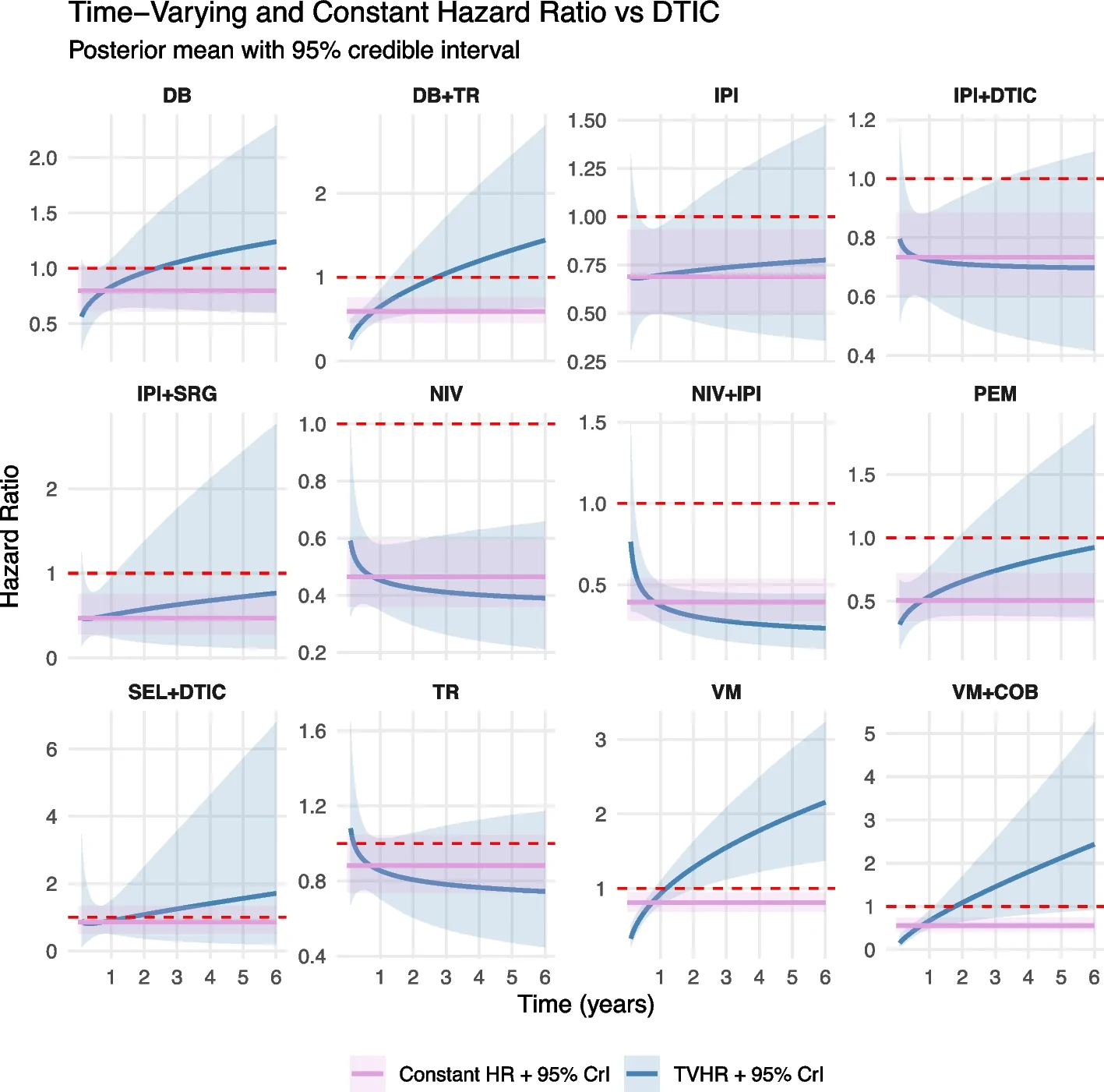
Melanoma network meta-analysis assuming time-varying and constant hazard ratios (HR), and associated 95% credible intervals (CrI)

Treatment comparisons were informed by single studies, with the exception of NIV + IPI versus IPI, which was informed by 2 studies. As a result, we present findings from fixed effect models as the primary network meta-analysis, which also allow comparison with alternative approaches to address time-varying treatment effects as reported in [17]. Pooled HRs and corresponding 95% credible intervals (CrI) obtained from the Cox proportional hazards models and TVHR Cox models are illustrated in Fig. 5. Table 2 presents the pooled HRs and mean treatment rankings from the network meta-analysis for each treatment compared to dacarbazine (DTIC), both under a constant hazard assumption and at one, two, and five years under a time-varying assumption. Of the 13 studies included, 9 (69%) were extrapolated beyond the observed period for an average extrapolation period of 2 years (SD: 1.34). Four studies (BREAK-3, CheckMate 067, Keynote 006, and Robert 2011) had an observed period of 5 of more years.

**Table 2:** Melanoma network meta-analysis - pooled constant & time-varying hazard ratios (HR), mean rank and associated 95% credible intervals (CrI) versus Dacarbazine (DTIC)

Treatment	Time (years)	HR	95% CrI	Mean rank	95% CrI
TR	Constant	0.88	0.74 to 1.04	10.98	8 to 13
	0.5	0.91	0.75 to 1.10	11.58	9 to 13
	1	0.86	0.71 to 1.03	9.93	7 to 13
	2	0.81	0.60 to 1.06	7.22	3 to 11
	5	0.75	0.47 to 1.15	5.79	3 to 10
IPI+DTIC	Constant	0.73	0.60 to 0.89	8.32	5 to 11
	0.5	0.74	0.60 to 0.90	9.41	7 to 12
	1	0.72	0.58 to 0.88	7.44	4 to 11
	2	0.71	0.52 to 0.94	5.82	3 to 10
	5	0.70	0.44 to 1.07	5.28	2 to 9
DB	Constant	0.80	0.61 to 1.03	9.54	6 to 13
	0.5	0.73	0.52 to 0.98	9.10	5 to 12
	1	0.83	0.63 to 1.08	9.51	6 to 13
	2	0.96	0.65 to 1.38	9.29	5 to 13
	5	1.19	0.61 to 2.10	8.84	4 to 12
DB+TR	Constant	0.59	0.46 to 0.76	5.54	2 to 8
	0.5	0.48	0.36 to 0.64	4.19	1 to 7
	1	0.65	0.48 to 0.85	5.83	3 to 9
	2	0.87	0.56 to 1.30	7.90	3 to 12
	5	1.32	0.62 to 2.49	9.55	4 to 13
VM	Constant	0.81	0.69 to 0.95	9.84	7 to 12
	0.5	0.66	0.55 to 0.80	8.10	5 to 11
	1	0.92	0.77 to 1.09	11.09	9 to 13
	2	1.28	0.99 to 1.63	12.32	10 to 13
	5	1.98	1.30 to 2.89	12.00	10 to 13
VM+COB	Constant	0.56	0.41 to 0.74	4.87	1 to 8
	0.5	0.41	0.28 to 0.59	2.64	1 to 7
	1	0.67	0.48 to 0.90	6.28	3 to 10
	2	1.09	0.66 to 1.70	10.24	5 to 13
	5	2.13	0.88 to 4.38	11.69	7 to 13
IPI	Constant	0.69	0.50 to 0.94	7.71	5 to 12
	0.5	0.68	0.47 to 0.96	8.56	6 to 12
	1	0.70	0.50 to 0.95	7.13	4 to 11
	2	0.72	0.46 to 1.07	6.25	4 to 11
	5	0.76	0.38 to 1.39	5.83	3 to 10
NIV	Constant	0.47	0.36 to 0.59	3.05	1 to 5
	0.5	0.49	0.37 to 0.63	4.49	2 to 7
	1	0.45	0.35 to 0.58	2.61	2 to 4
	2	0.43	0.30 to 0.59	2.44	2 to 4
	5	0.40	0.23 to 0.65	2.54	2 to 4
NIV+IPI	Constant	0.39	0.28 to 0.54	1.42	1 to 3
	0.5	0.46	0.31 to 0.65	3.55	1 to 7
	1	0.37	0.26 to 0.51	1.26	1 to 2
	2	0.31	0.19 to 0.46	1.17	1 to 2
	5	0.24	0.12 to 0.45	1.18	1 to 2
PEM	Constant	0.51	0.35 to 0.72	4.02	2 to 7
	0.5	0.45	0.29 to 0.66	3.22	1 to 7
	1	0.54	0.36 to 0.77	4.08	2 to 8
	2	0.66	0.39 to 1.04	5.11	3 to 10
	5	0.87	0.38 to 1.73	6.86	3 to 12
IPI+SRG	Constant	0.47	0.28 to 0.76	3.21	1 to 8
	0.5	0.47	0.27 to 0.78	3.88	1 to 9
	1	0.51	0.24 to 0.95	3.68	1 to 11
	2	0.57	0.18 to 1.38	4.25	1 to 13
	5	0.72	0.12 to 2.43	4.89	1 to 13
SEL+DTIC	Constant	0.85	0.51 to 1.35	9.83	4 to 13
	0.5	0.82	0.43 to 1.41	9.68	3 to 13
	1	0.90	0.51 to 1.50	9.77	3 to 13
	2	1.08	0.36 to 2.50	8.86	2 to 13
	5	1.55	0.21 to 5.73	8.21	1 to 13

Under the constant HR assumption, the combination of nivolumab plus ipilimumab (NIV+IPI) demonstrated the greatest efficacy in terms of overall survival, yielding a pooled HR of 0.39 (95% CrI: 0.28 to 0.54) and the highest mean rank of 1.42 (95% CrI: 1 to 3). Nivolumab monotherapy (NIV) and pembrolizumab (PEM) also showed substantial benefit, with pooled HRs of 0.47 (95% CrI: 0.36 to 0.59) and 0.51 (95% CrI: 0.35 to 0.72), respectively. Several other treatments including ipilimumab (IPI) monotherapy, ipilimumab plus dacarbazine (IPI+DTIC), and vemurafenib plus cobimetinib (VM+COB), demonstrated statistically credible reductions in the hazard of death relative to DTIC, as evidenced by 95% credible intervals excluding unity.

**Fig. 6:**
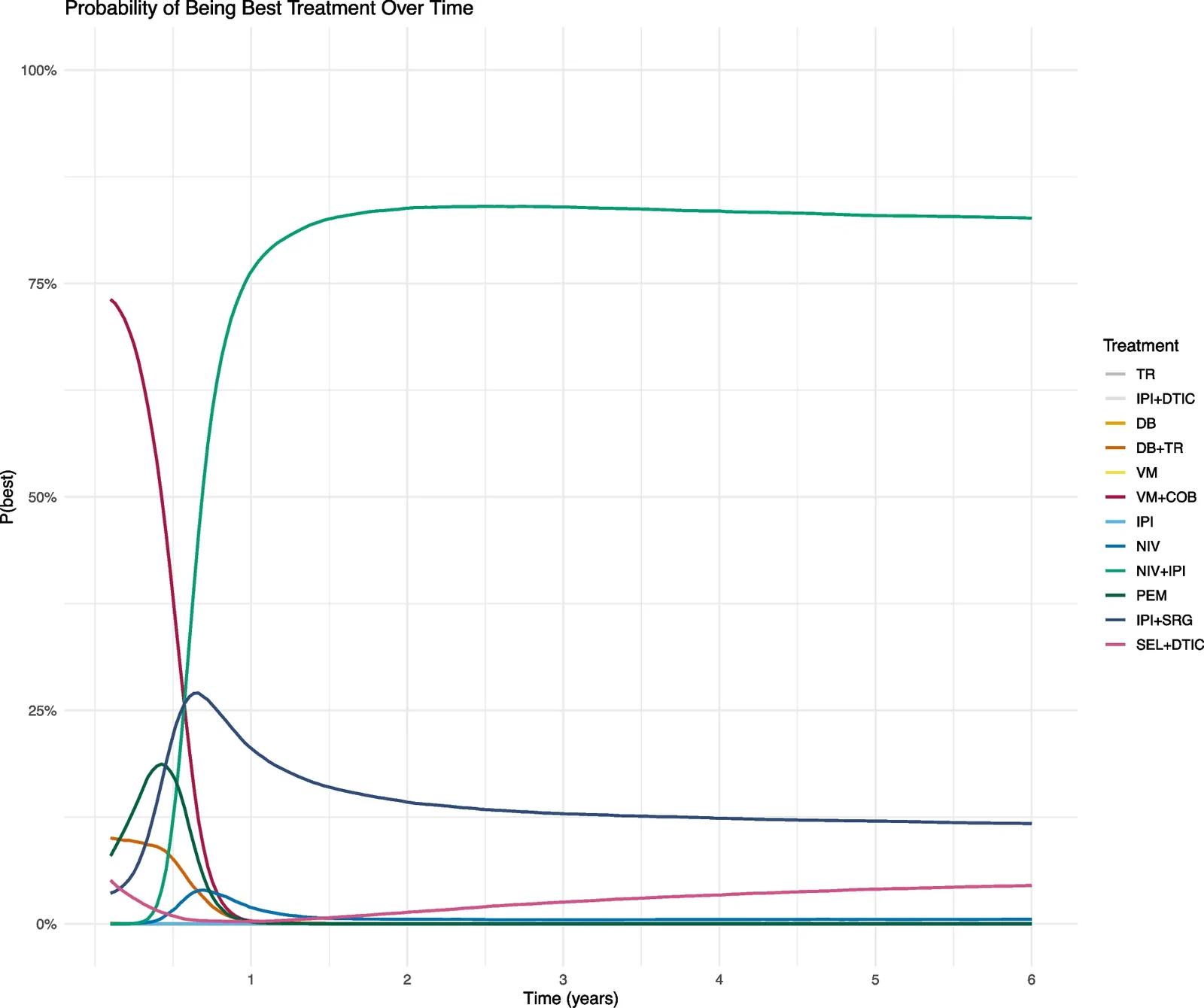
Melanoma network meta-analysis - probability best over time using a TVHR NMA model

The time-varying analyses revealed important heterogeneity in treatment effects over time. Figures 6 and B.3 (see Additional File B) illustrate how the probability that each treatment was the best overall changes over time. However, it should be noted that rank probabilities can be affected by the number of studies per comparison and the overall network geometry [32]. NIV+IPI maintained consistent superiority from month 7 onwards, with an improving HR from 0.46 (95% CrI: 0.31 to 0.65) at 6 months to 0.24 (95% CrI: 0.12 to 0.45) at five years, and retaining the highest mean rank from month 7 (Table 2 & Fig. B.3, see Additional File B). NIV demonstrated a similarly durable and improving effect, with the pooled HR falling to 0.40 (95% CrI: 0.23 to 0.65) at five years. IPI-based regimens (IPI, IPI+DTIC, IPI+SRG) showed broadly stable pooled HRs over time, suggesting a sustained but non-improving treatment effect (Fig. 5).

In contrast, several targeted therapy combinations exhibited waning treatment effects over time (Fig. 5). VM+COB showed a pronounced pattern, with the highest probability of being the best treatment at earlier time points (Figs. 6 & B.3, see Additional File B) but with rising pooled HRs from 0.56 (95% CrI: 0.41 to 0.74) at the constant time point to 2.13 (95% CrI: 0.88 to 4.38) at five years. VM alone followed a similar trajectory, with the pooled HR increasing to 1.98 (95% CrI: 1.30 to 2.89) at five years, suggesting that VM alone may become inferior relative to DTIC over longer time horizons. However, this estimate is based on a log-linear extrapolation far beyond the observed data (2.5 years) and should be interpreted with caution. Dabrafenib based treatments (DB+TR, DB) also demonstrated waning effects (Fig. 5), for example DB+TR had an estimated pooled HR at 6 months of 0.48 (95% CrI: 0.36 to 0.64), and crossing unity by five years (1.32, 95% CrI: 0.62 to 2.49), with its mean rank deteriorating from 4.19 (95% CrI: 1 to 7) to 9.55 (95% CrI: 4 to 13) (Table 2). Credible interval widths generally increased at longer time points across all treatments, reflecting greater uncertainty in long-term projections, and this was particularly pronounced for treatments with fewer long-term data, such as IPI+SRG and SEL+DTIC (Table 2). All values of $\hat{R}<$ 1.01, and ESS >1000 per parameter, suggesting that the MCMC chains have converged to a common stationary distribution with reliable posterior inference. The results were robust to assuming a random effects model (Figs. B.4, B.5 & B.6, see Additional File B). As a result of the limited data to inform estimation of the variability between studies, there is considerable uncertainty in the resulting estimated HRs (Fig. B.4, see Additional File B).

## Discussion

This paper adds to the literature a bivariate (pairwise) and multivariate (network) Bayesian meta-analysis framework for a treatment-log(time) interaction obtained from standard Cox models. This approach yields an interpretable time-varying hazard ratio with appropriately propagated uncertainty, and is estimable from routinely reported Cox model outputs (if a TVHR model has been fitted and covariance matrix reported) or reconstructed from published Kaplan-Meier estimates, and integrates directly into hazard-ratio-based economic decision models without requiring a change in modelling paradigm. More broadly, the TVHR meta-analysis approach presented here, which assumes a log-linear effect with time, offers a pragmatic and accessible alternative to more complex methods such as fractional polynomial models [3] for relaxing the proportional hazards assumption. While retaining relative simplicity, this framework can readily be incorporated into cost-effectiveness models (by allowing the TVHR to be applied to the baseline hazard in a target population), making it well-suited to HTA contexts. Where more complex hazard relationships are anticipated, the linear treatment-log(time) interaction term in (2) could itself be replaced with a spline basis in log(time) (e.g. restricted cubic or M-splines), retaining the same two-stage Cox-model then bivariate/multivariate-synthesis structure described in Methods section, but allowing a more flexible, non-monotonic time-varying HR. This is distinct from existing fully non-parametric approaches such as M-spline NMA models applied to the baseline hazard within a one-stage framework [9]; rather, it is a proposed extension of the present TVHR framework’s interaction term, retaining its two-stage, hazard-ratio-scale structure.

In practice, the trials contributing to a pairwise or network meta-analysis are often relatively short in duration, so that complex, non-monotonic hazard trajectories are frequently not observed within the follow-up period itself. Over this observed period, a log-linear effect in time will often provide a reasonable approximation to the underlying time-varying treatment effect. This does not, however, imply that the log-linear form remains appropriate when extrapolated over the lifetime horizons typically required in cost-effectiveness analysis, where the hazard ratio trajectory implied by the model may diverge substantially from the true long-term effect. We therefore recommend that log-linear extrapolations be treated as one plausible scenario rather than a default assumption, and that they are checked for clinical plausibility over the extrapolated period (as any extrapolation method should be) and, where possible, validated against longer-term or real-world data. Where individual patient-level covariate information is available, the framework could be further extended by adopting a Multi-Level Network Meta-Regression (ML-NMR) approach [33], enabling more nuanced exploration of treatment effect heterogeneity.

A two-stage approach has been employed throughout. This is a practical necessity in many applied settings since covariate information is frequently unavailable, particularly where Kaplan-Meier estimates have been digitised [4], and thus the additional benefits of a one-stage approach are limited in these circumstances.

From a reporting and validation perspective, a TVHR approach aligns naturally with how results are commonly communicated in the clinical literature, where trials typically report HRs rather than RMST. This makes the approach particularly useful for checking the face validity of pairwise or network meta-analysis results intended to inform decision-making, as outputs remain interpretable to both clinical and HTA audiences.

We emphasise that the TVHR approach itself generates no new clinical evidence; rather, it changes how the time-varying nature of treatment effects already present in trial data is synthesised and subsequently applied to inform long-term extrapolation within economic decision models. Its clinical relevance therefore lies in the substantive findings it enables. For example, the durable and improving benefit of nivolumab plus ipilimumab and nivolumab monotherapy relative to dacarbazine, against the waning and potentially reversing benefit of dabrafenib- and vemurafenib-based regimens, rather than in the statistical method per se.

The TVHR framework offers a principled basis for extrapolating treatment effects beyond the observed follow-up period, with appropriate propagation of uncertainty. This is of particular relevance in HTA, where long-term survival extrapolation is routinely required [34]. The importance of capturing time-varying treatment effects is underscored by the emergence of novel immunotherapy combinations in oncology, such as nivolumab plus ipilimumab (NIV+IPI), which exhibit a decreasing HR over time, and one that has profound implications for long-term survival extrapolation and cost-effectiveness conclusions. As such technologies become more prevalent, methods that can appropriately characterise and extrapolate evolving treatment effects are becoming increasingly important for robust decision-making. Critically, the question of treatment waning is frequently raised in HTA submissions, often with arbitrary scenario analyses applied to explore the impact of changing treatment effects over time [11]. The TVHR approach provides a data-driven basis for exploring such scenarios, grounding long-term projections in the observed trajectory of treatment effects and offering a more transparent and defensible method for characterising uncertainty around treatment waning assumptions and the implications that this might have for guidance.

Several alternative approaches exist for handling non-proportional hazards in the context of meta-analysis and network meta-analysis. Fractional polynomial models [3] offer a flexible parametric framework for capturing complex hazard functions and have been widely used in HTA [35]. However, they can be difficult to implement within network meta-analysis [36], particularly when heterogeneity in the shape of the hazard function exists across trials, and the resulting models can be challenging to incorporate into economic decision models in a transparent and intuitive manner. By contrast, the treatment-log(time) interaction approach assumes a simpler log-linear relationship with log(time), which, whilst less flexible, is more transparent and more readily communicated to a non-specialist audience.

Restricted mean survival time (RMST) based approaches offer a useful alternative that avoids the proportional hazards assumption entirely, and provide an absolute and easily interpretable summary measure of treatment benefit [37]. However, trials typically report HRs rather than RMST estimates, and RMST-based network meta-analysis results can be less straightforward to incorporate into cost-effectiveness models or to validate against other published trial results. The TVHR approach, by retaining the HR as the effect measure, aligns more naturally with how evidence is reported, applied (to a target baseline population), and appraised in HTA.

Piecewise exponential models represent another option, allowing the baseline hazard and treatment effects to vary across pre-specified time intervals [38]. Whilst flexible, the choice of cut-points can be arbitrary and results sensitive to their specification [39]. The log-linear interaction approach developed here avoids this by imposing a smooth, continuous relationship between the treatment effect and time, which is both more parsimonious and more amenable to extrapolation.

Bayesian parametric IPD network meta-analysis approaches, including Weibull, Gompertz, log-normal, log-logistic, gamma, and generalised gamma, including multivariate parameterisation of the shape and scale parameters, are alternative approaches [6]. These approaches capture the time-varying nature directly through the choice of parametric distribution. In HTA, particularly for cost-effectiveness modelling, a common approach is to fit a parametric survival model to a reference treatment arm and apply HRs to obtain survival curves for comparator treatments, or jointly estimate the baseline hazard and treatment effects within a single parametric distributional family [40]. The former lends itself to a TVHR approach, whereas the latter may prefer a parametric network meta-analysis approach. However, the parametric network meta-analysis approach constrains the survival curves to belong to the same parametric family, which may not be the most appropriate distribution across all treatments included in the network. The TVHR approach separates estimation of the baseline survival models and the synthesis of relative treatment effects, which offers a more straight-forward but flexible implementation in economic models.

A flexible network meta-analysis model for time-to-event outcomes using an M-spline approach on the baseline hazard has been proposed [9], with a weighted random walk prior distribution that provides shrinkage, designed specifically to handle complex hazard functions. The M-spline approach is a highly flexible Bayesian one-step model that places a smooth non-parametric prior distribution on the baseline hazard, allowing it to capture arbitrary departures from proportionality, including delayed treatment effects, crossing survival curves, or non-monotone HRs, without specifying a functional form for the time-varying HR. Our approach, by contrast, assumes a specific parametric structure, that is a linear relationship between the log-HR and log(time) within a Cox model, yielding a time-varying HR that varies as a function of log(time). This imposes a more restrictive but more interpretable form. However, as discussed above, to capture more complex hazard shapes, where required, the linear log-time interaction of the TVHR approach can be generalised to a spline function in log(time), and the bivariate synthesis extended to higher dimensions.

Efficient computational strategies have also recently been proposed for exploring time-varying hazard ratio structures at scale, by re-expressing fractional-polynomial and piecewise-constant NMA models as generalised linear models with time-varying covariates. A large number of candidate models can be screened rapidly in a frequentist framework using AIC/BIC before the preferred model is re-estimated in a Bayesian framework to propagate uncertainty for decision-making [41]. Similarly, one-step Bayesian parametric NMA models using the exact likelihood have been developed that can accommodate time-varying treatment effects directly, avoiding some of the efficiency losses associated with a two-stage approach [6]. These developments are complementary to the TVHR framework presented here in that they offer greater flexibility in the shape of the time-varying hazard ratio and, in the case of one-step approaches, a more statistically efficient synthesis, but at the cost of additional modelling complexity and, for the GLM-based approaches, a reliance on model-selection criteria that are less familiar to a non-specialist HTA audience compared to a simple hazard ratio.

### Limitations

Whilst the treatment-log(time) interaction approach offers a pragmatic and accessible framework for capturing time-varying treatment effects, several limitations should be acknowledged. First, the assumption of a log-linear relationship between the HR and log(time), whilst often reasonable in the context of relatively short-term RCTs, may not adequately capture more complex or non-monotonic hazard trajectories in all settings, for example in Real World Evidence (RWE) with longer follow-up [42]. Although extensions incorporating spline terms on the interaction with log-time can address this, such models require greater data to support them and may be difficult to justify in sparse networks.

Secondly, the two-stage approach adopted here, whilst practical, introduces some efficiency loss relative to a one-stage model, particularly where there is substantial between-trial heterogeneity [43]. This limitation is compounded in settings where IPD are unavailable and Kaplan-Meier estimates have been digitised, as this process introduces additional uncertainty that is not always fully propagated through subsequent analyses [4, 44].

Thirdly, whilst the framework allows for extrapolation of time-varying treatment effects beyond the observed follow-up period, such projections remain inherently uncertain, and the plausibility of long-term extrapolations, including (constrained) spline-based models [45], should always be assessed using external data and/or in consultation with patient and clinical experts [46].

Fourthly, in the gastric cancer example, one of the five studies contributing evidence of non-proportional hazards (Study 9) showed a treatment-log(time) interaction in the opposite direction to the remaining four; because the bivariate synthesis pools across a small number of informative studies, individual discordant studies can materially affect the pooled interaction term, and leave-one-out sensitivity analyses are recommended in applied use.

Fifthly, in the present two-stage implementation the TVHR approach could not be formally compared with a standard constant-HR NMA using the Deviance Information Criterion (DIC) or any commonly used model fit statistics depending on the likelihood. This is because the first-stage Cox models for the constant-HR and TVHR approaches are fitted via partial likelihood outside the Bayesian synthesis, and the second-stage univariate and bivariate/multivariate meta-analysis models use different dimensional summary data (one coefficient per study versus two), so their deviances and effective numbers of parameters are not directly comparable via a shared DIC. Two more tractable alternatives are available within the present framework: (i) the posterior probability that the pooled treatment-log(time) interaction is non-zero (reported here as 0.97 for the gastric cancer example), which provides an informal, study-level indication of whether allowing for a TVHR is warranted; and (ii) visual and predictive comparison of the fitted HR trajectories against the observed Kaplan-Meier estimates and Schoenfeld residuals for each study. A formal DIC-based (or WAIC-based) comparison against constant-HR and against more flexible time-varying models (e.g. fractional polynomial, M-spline) would require a fully Bayesian one-stage implementation in which all models share a common likelihood for the individual (or pseudo-individual) participant data; this is identified as a priority for future work.

Whilst the TVHR model assumes a time-varying HR for each study, in practice some studies may have a HR that is close to being constant, which will be propagated through the model through a treatment-log(time) interaction term close to 0. This is demonstrated for the IPI+DTIC treatment effect, which is informed by a single study, where the HRs obtained from the TVHR approach are similar to the constant HR model (Fig. 5). In situations where studies report only the HRs and confidence intervals (i.e., no Kaplan-Meier plots, which can be digitised), a TVHR model can not be estimated but they could nevertheless be included by extending the TVHR model to have a hierarchical structure. For example, the study-specific treatment-log(time) parameter could be assumed to be exchangeable with treatments of the same class [47, 48].

### Knowledge gaps

Several knowledge gaps remain. First, there is no formal, objective comparison of the TVHR approach against alternative methods for handling non-proportional hazards (e.g. fractional polynomial, Royston-Parmar, piecewise-exponential, M-spline or RMST-based models) in terms of predictive accuracy, extrapolation performance, or impact on downstream cost-effectiveness estimates; existing comparative work has largely been undertaken by developers of individual methods rather than by independent, impartial parties, and there is correspondingly little consensus-based guidance on when one approach should be preferred over another. Second, the relative performance of a two-stage TVHR synthesis versus a fully Bayesian one-stage implementation, in terms of both statistical efficiency and the propagation of digitisation uncertainty when Kaplan-Meier estimates are reconstructed, has not been formally quantified. Third, it remains unclear how well a log-linear treatment-log(time) interaction extrapolates beyond the observed follow-up period relative to more flexible (e.g. spline-based) time-varying structures (which can also be influenced by the tails of the observed distribution), particularly over the long (often lifetime) horizons typically required in cost-effectiveness analysis. This is likely to depend on the mechanism generating non-proportionality (e.g. delayed treatment effect, waning effect, crossing hazards) in ways that are not yet well characterised.

### Future directions

Future work should prioritise: (i) head-to-head, impartial comparisons of the TVHR approach against fractional polynomial, Royston-Parmar/M-spline, piecewise-exponential and RMST-based synthesis methods across a range of non-proportional hazards mechanisms and network structures, ideally using simulation studies with a known data-generating process, to inform objective method-selection guidance for HTA; (ii) development and validation of a fully Bayesian one-stage TVHR model, which would allow formal comparison with one-stage alternatives via information criteria such as DIC/WAIC and avoid the potential efficiency losses of the two-stage approach; (iii) extension of the linear treatment-log(time) interaction to a spline-based interaction term (e.g. restricted cubic or M-splines in log-time) within the same bivariate/multivariate synthesis framework, for use where a log-linear relationship is judged inadequate, together with guidance on knot selection in sparse network settings; (iv) extension to a hierarchical structure in which study-specific treatment-log(time) interactions are exchangeable across treatments of the same class [47, 48], to allow inclusion of studies reporting only a constant HR; and (v) empirical evaluation of TVHR extrapolations against long-term follow-up or real-world evidence, to establish how reliably a log-linear (or spline-extended) hazard ratio trend estimated from short/medium-term trial data predicts observed long-term treatment effects.

## Conclusion

In the two motivating examples presented, a treatment-log(time) interaction within a Cox model, synthesised using a bivariate (pairwise) or multivariate (network) Bayesian meta-analysis, provided an interpretable time-varying hazard ratio that revealed patterns not captured under a constant-HR assumption, including an apparent loss of chemotherapy benefit for progression-free survival beyond approximately one year in advanced gastric cancer, and a durable, improving benefit of nivolumab-based immunotherapy against a waning benefit of dabrafenib- and vemurafenib-based regimens in advanced BRAF-mutated melanoma. Because the resulting estimates remain on the hazard ratio scale, they can be applied directly to a baseline hazard within a cost-effectiveness model in the same way as a conventional constant HR, offering a more transparent basis for exploring treatment-effect waning than the arbitrary scenario analyses often used in HTA submissions. Incorporating a treatment-log(time) interaction within a meta-analysis or network meta-analysis of time-to-event outcomes provides a simple and intuitive approach to relaxing the proportional hazards assumption where it is violated in one or more included studies.

## Supplementary Information

Supplementary Material 1. [49, 50]

## Data Availability

All data used are available from the cited publications and available in the NMA-TVHR GitHub repository, https://github.com/r-k-owen/NMA-TVHR.

## Abbreviations

AIC: Akaike Information Criterion
BIC: Bayesian Information Criterion
COB: Cobimetinib
CrI: Credible Interval
DB: Dabrafenib
DIC: Deviance Information Criterion
DTIC: Dacarbazine
ESS: Effective Sample Size
GLM: Generalised Linear Model
HR: Hazard Ratio
HTA: Health Technology Assessment
IPD: Individual Participant Data
IPI: Ipilimumab
MCMC: Markov Chain Monte Carlo
MVN: Multivariate Normality
NIV: Nivolumab
NMA: Network Meta-Analysis
OS: Overall Survival
PEM: Pembrolizumab
PFS: Progression-Free Survival
RCT: Randomised Controlled Trial
RMST: Restricted Mean Survival Time
RWE: Real World Evidence
SD: Standard Deviation
SEL: Selumetinib
SoC: Standard of Care
SRG: Sargramostin
TR: Trametinib
TRL: Tremelimumab
TTE: Time-to-event
TVHR: Time-varying Hazard Ratio
VM: Vemurafenib
WAIC: Watanabe-Akaike or Widely Applicable Information Criterion
