# Supplementary material for: Meta-analysis and network meta-analysis of time-to-event outcomes with non-proportional hazards: a Bayesian time-varying hazard ratio approach
Source: BMC Med Res Methodol. 2026 Aug 4;26:234. doi: 10.1186/s12874-026-02963-5 (PMC13721545; doi:10.1186/s12874-026-02963-5)
Supplement: Supplementary file 1 [file 12874_2026_2963_MOESM1_ESM.pdf]

Additional File A Pairwise meta-analysis - Advanced Gastric Cancer

Time (Years)	HR	95% CrI
0.5	0.82	0.75 to 0.90
1.0	0.88	0.78 to 0.99
1.5	0.92	0.78 to 1.06
2.0	0.94	0.79 to 1.11
2.5	0.96	0.79 to 1.16
3.0	0.98	0.79 to 1.20
3.5	0.99	0.79 to 1.23

Table A.1 Gastric cancer meta-analysis - time-varying hazard ratios (HR) and associated 95% credible intervals (CrI) at specific time points for chemotherapy compared to standard of care estimated using Half-Normal(0,0.5) prior distribution for τ_2 & MCMC.

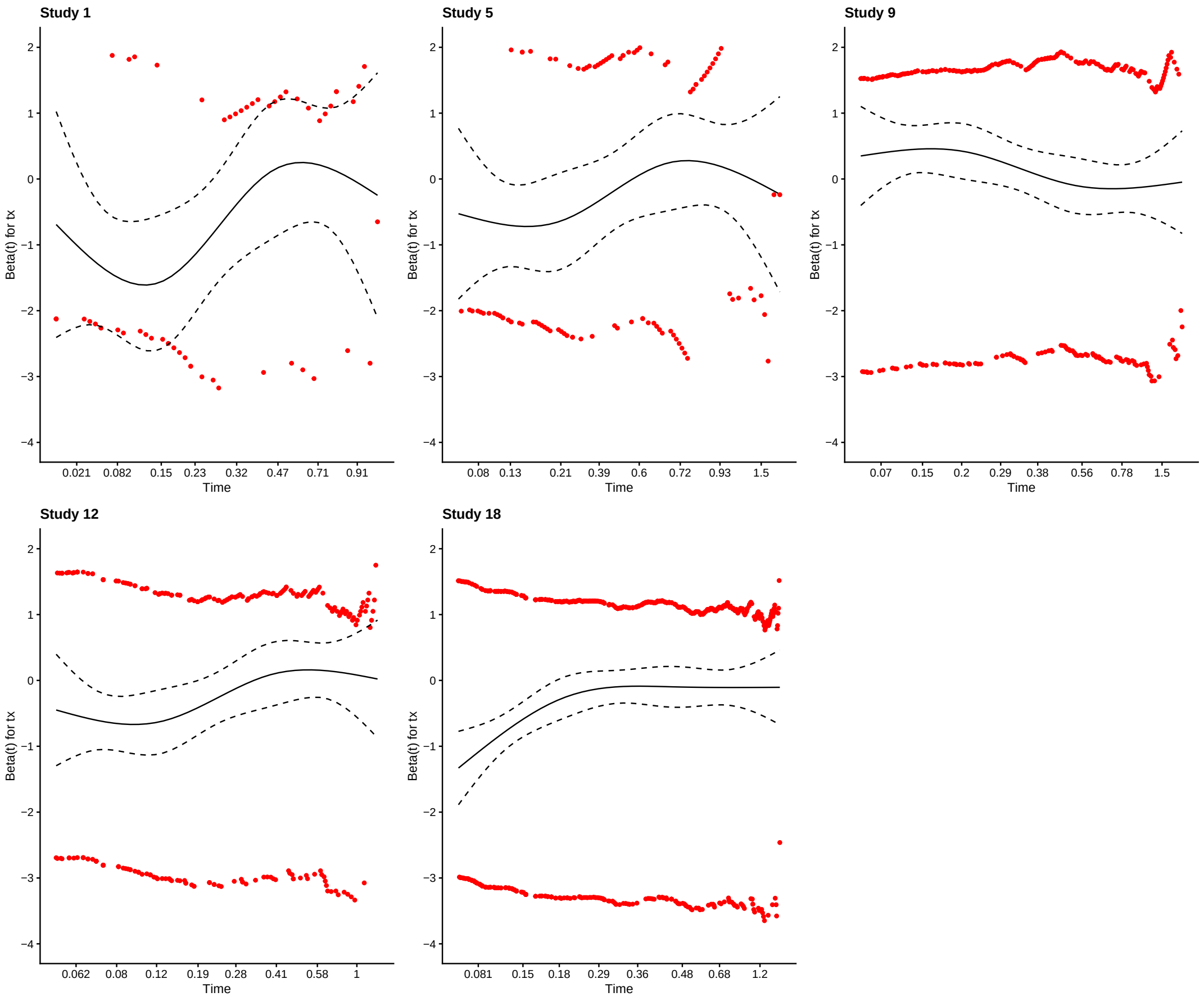

Fig. A.1 Gastric cancer meta-analysis - Schoenfeld Residual Plots for studies with non-proportional hazards.

Study	logHR	SE	(common)	(random)	IV, Fixed + Random, 95% CI	IV, Fixed + Random, 95% CI
Study 1	-0.6498	0.2693	1.6%	2.6%	0.52 [0.31; 0.89]	
Study 2	-0.5206	0.2737	1.5%	2.6%	0.59 [0.35; 1.02]	
Study 3	-0.1230	0.1854	3.3%	4.4%	0.88 [0.61; 1.27]	
Study 4	-0.2760	0.1398	5.8%	6.1%	0.76 [0.58; 1.00]	
Study 5	-0.2386	0.1920	3.1%	4.3%	0.79 [0.54; 1.15]	
Study 6	-0.4529	0.3543	0.9%	1.7%	0.64 [0.32; 1.27]	
Study 7	-0.0272	0.1712	3.9%	4.9%	0.97 [0.70; 1.36]	
Study 8	-0.0822	0.2125	2.5%	3.7%	0.92 [0.61; 1.40]	
Study 9	0.1572	0.1100	9.4%	7.5%	1.17 [0.94; 1.45]	
Study 10	-0.1887	0.1463	5.3%	5.8%	0.83 [0.62; 1.10]	
Study 11	-0.7293	0.1927	3.1%	4.2%	0.48 [0.33; 0.70]	
Study 12	-0.2532	0.1263	7.1%	6.7%	0.78 [0.61; 0.99]	
Study 13	-0.1556	0.1137	8.8%	7.3%	0.86 [0.68; 1.07]	
Study 14	-0.6399	0.1794	3.5%	4.6%	0.53 [0.37; 0.75]	
Study 15	-0.2111	0.1015	11.0%	7.9%	0.81 [0.66; 0.99]	
Study 16	-0.0922	0.1749	3.7%	4.8%	0.91 [0.65; 1.28]	
Study 17	-0.0850	0.2219	2.3%	3.5%	0.92 [0.59; 1.42]	
Study 18	-0.3247	0.0809	17.3%	9.0%	0.72 [0.62; 0.85]	
Study 19	-0.3926	0.2035	2.7%	3.9%	0.68 [0.45; 1.01]	
Study 20	-0.4009	0.1849	3.3%	4.5%	0.67 [0.47; 0.96]	
**Total (common effect, 95% CI)**			**100.0%**	.	**0.79 [0.74; 0.85]**	
**Total (random effect, 95% CI)**			.	**100.0%**	**0.78 [0.71; 0.86]**	

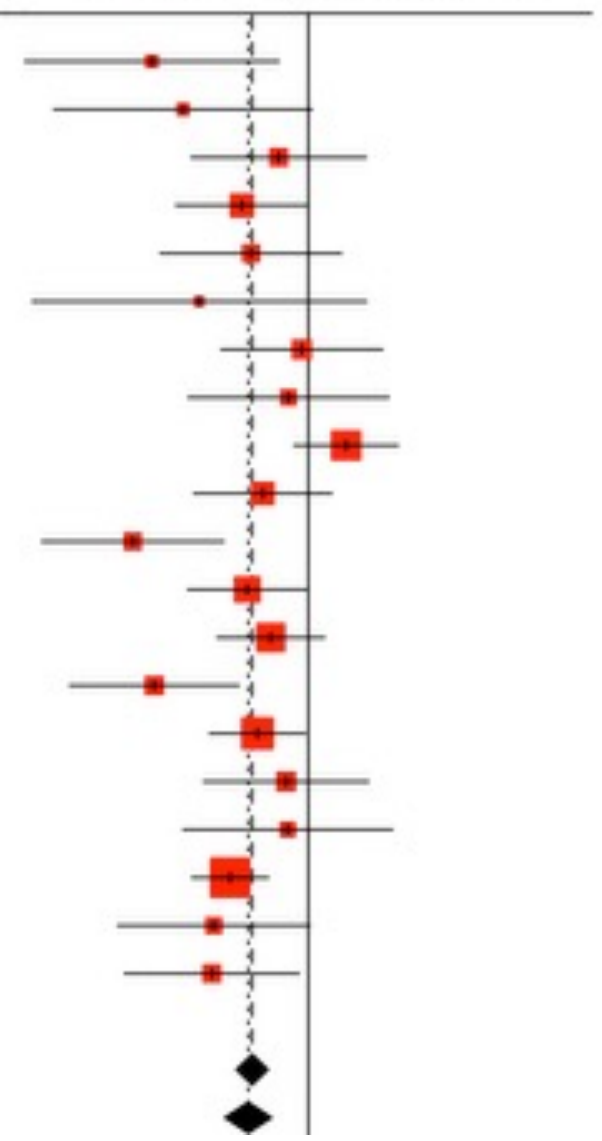

Fig. A.2 Gastric cancer meta-analysis - two-stage meta-analysis of Hazard Ratios (HRs).

Figure A.2 displays the results of undertaking a frequentist 2-stage meta-analysis (using REML in the `meta` package [49] in `R` [19] of the log-hazard ratios (and associated standard errors) obtained by applying (1) to each study. The overall pooled HR is 0.78 with 95% CI (0.71 to 0.86) with statistically significant evidence against an assumption of homogeneity (P=0.01).

A 1-stage frequentist analysis of the IPD from all 20 studies, and in which each trial is assumed to have a study-specific constant HR and an independent baseline together with a random treatment effect (implemented using `coxme` in `R` [19, 43, 50]), yielded a pooled HR of 0.83 with 95% CI (0.74 to 0.92).

Additional File B Network meta-analysis - Advanced BRAF-mutated Melanoma

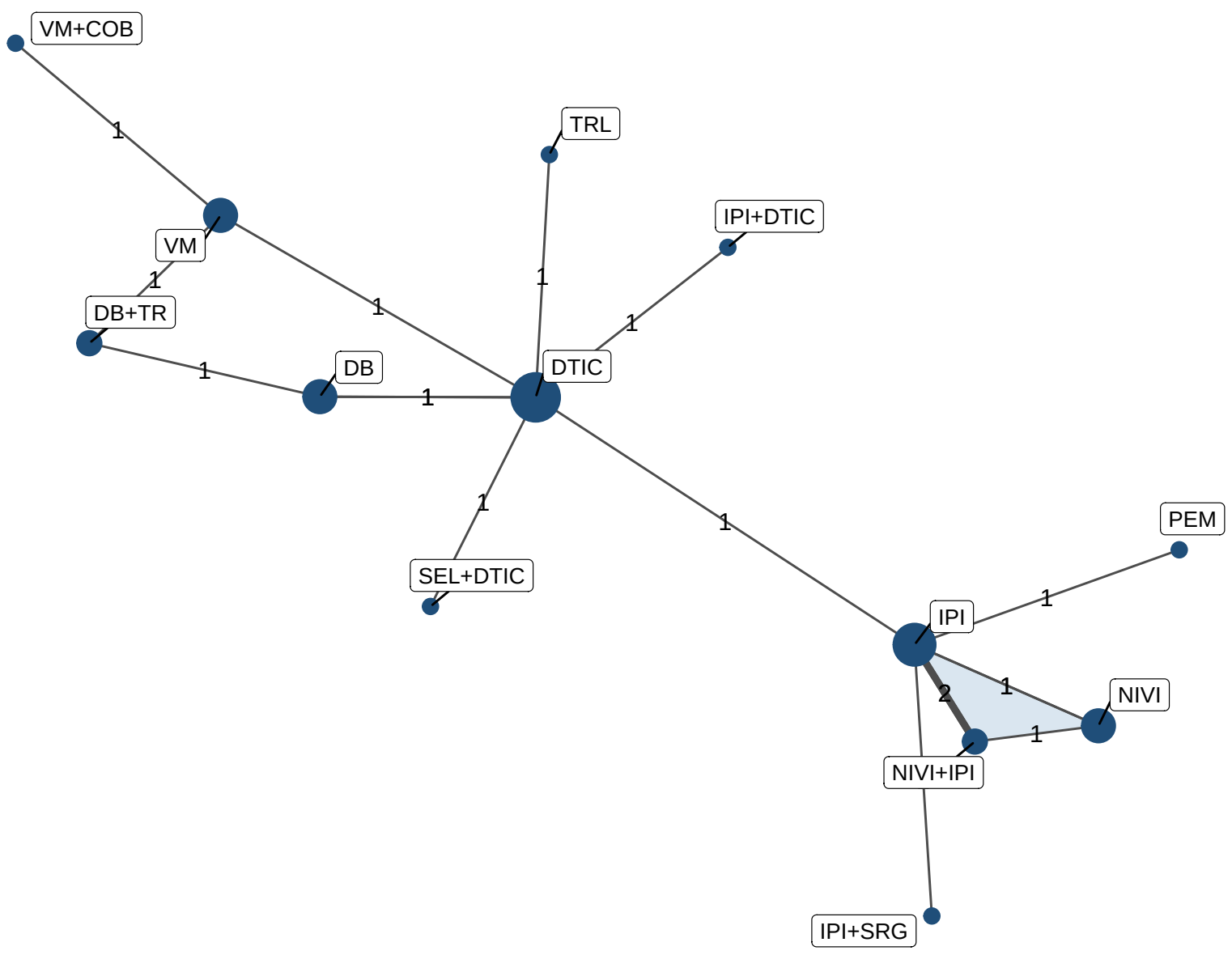

Fig. B.1 Advanced BRAF-mutated melanoma NMA - Evidence network plot.

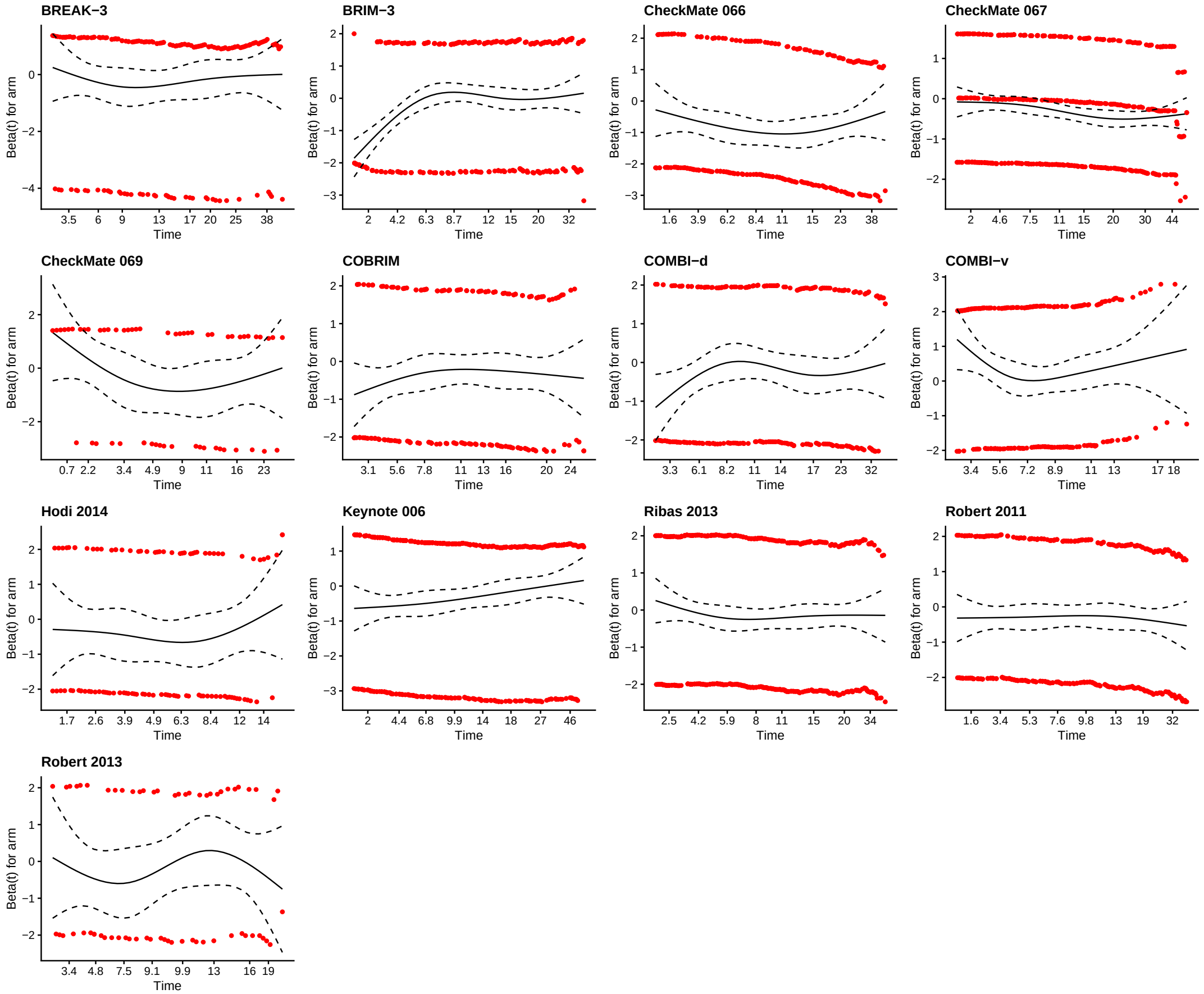

Fig. B.2 Advanced BRAF-mutated melanoma NMA - Schoenfeld Residual Plots.

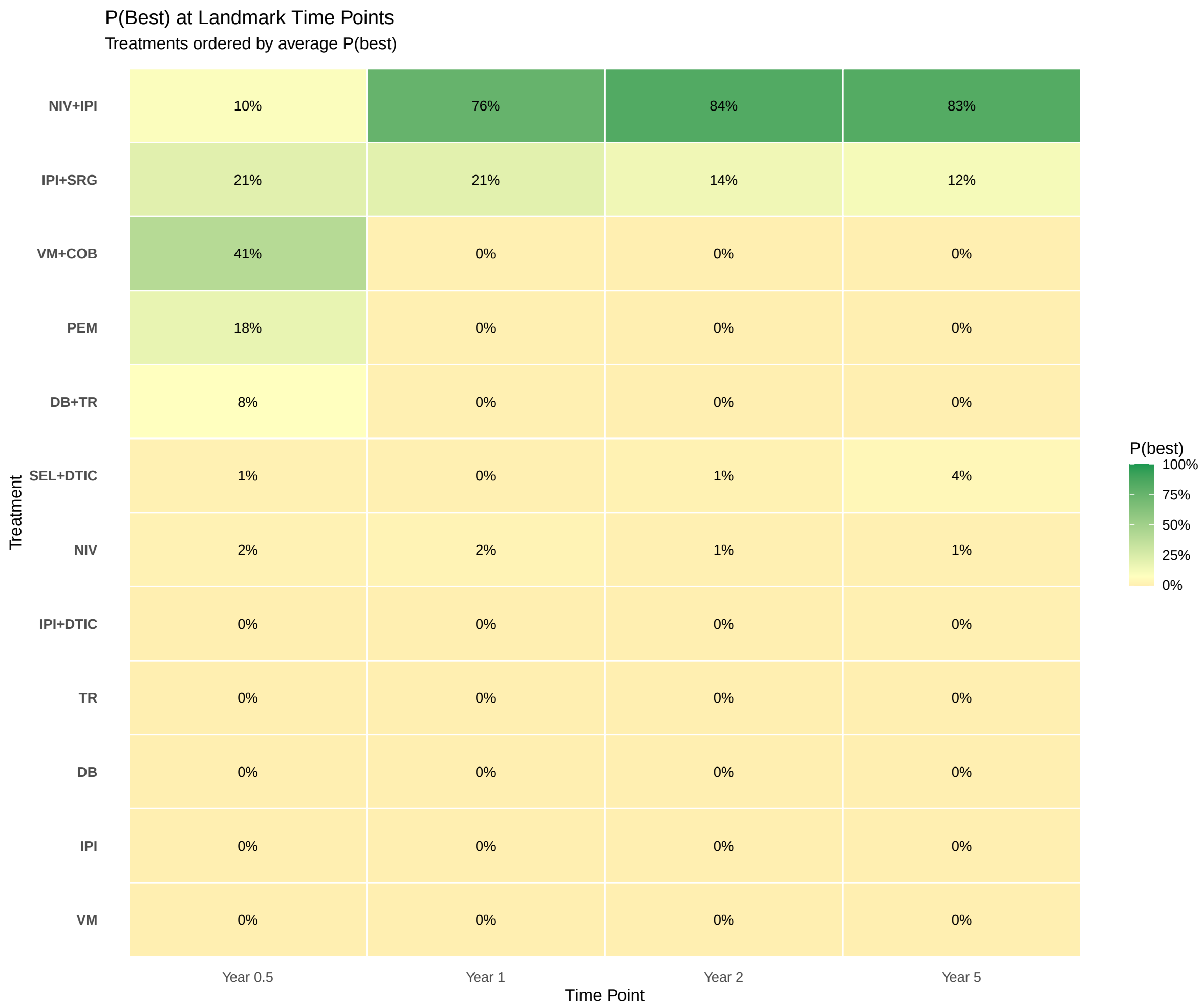

Fig. B.3 Melanoma network meta-analysis - heatmap of probability best at landmark time points.

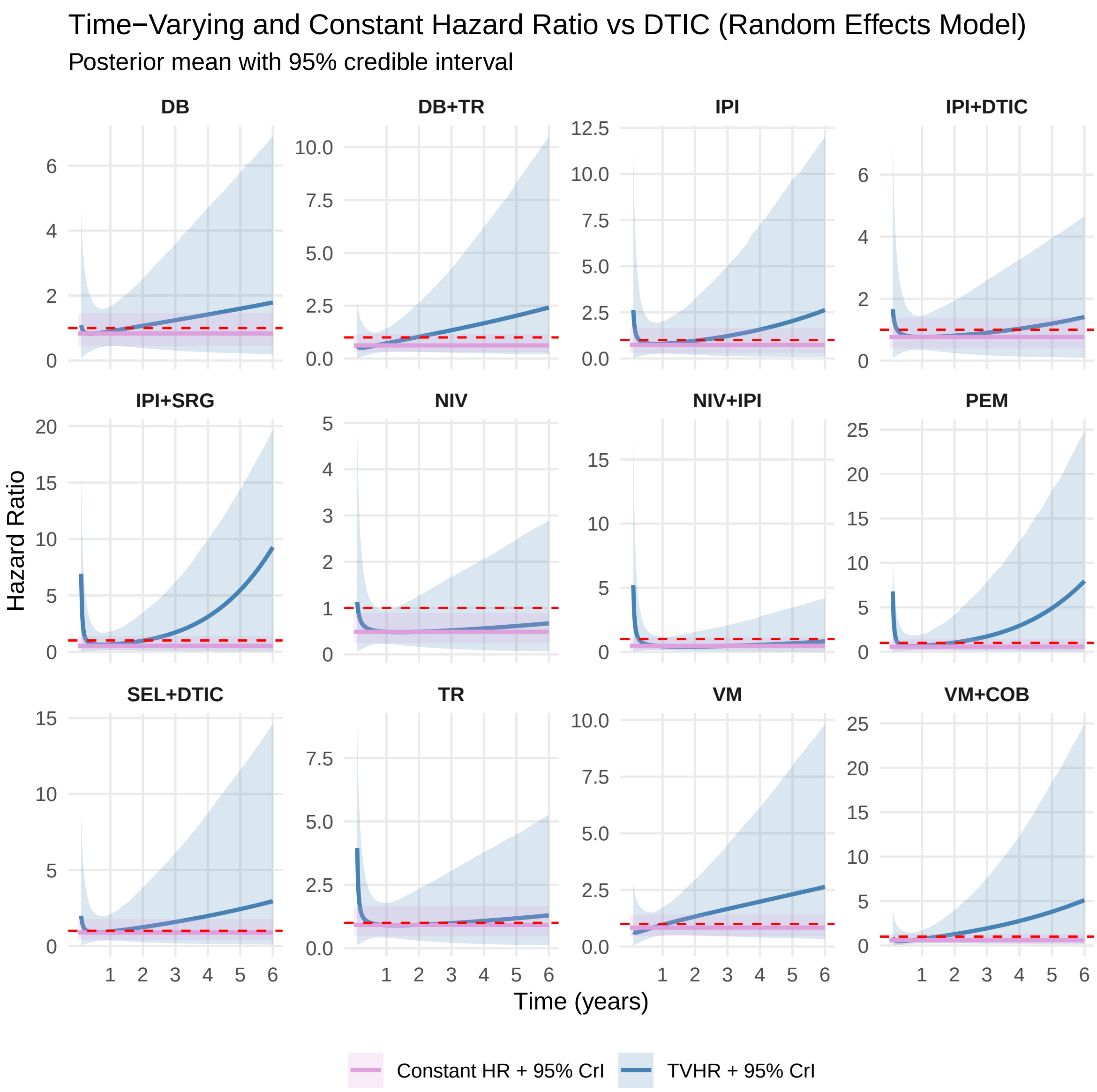

Fig. B.4 Melanoma network meta-analysis assuming time-varying and constant hazard ratios (HR) assuming random effects, and associated 95% credible intervals (CrI).

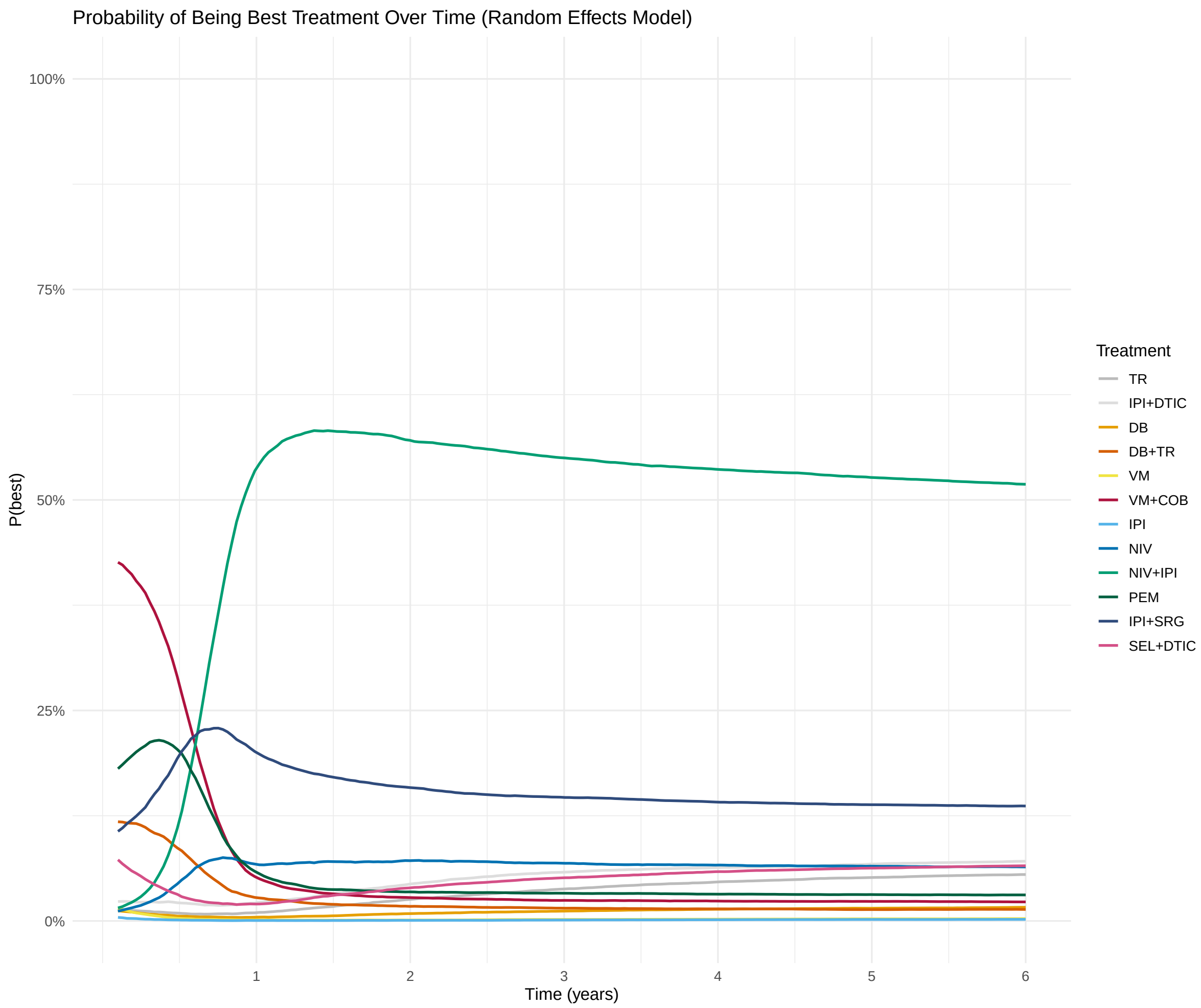

Fig. B.5 Melanoma network meta-analysis - probability best over time using a TVHR NMA model assuming random effects.

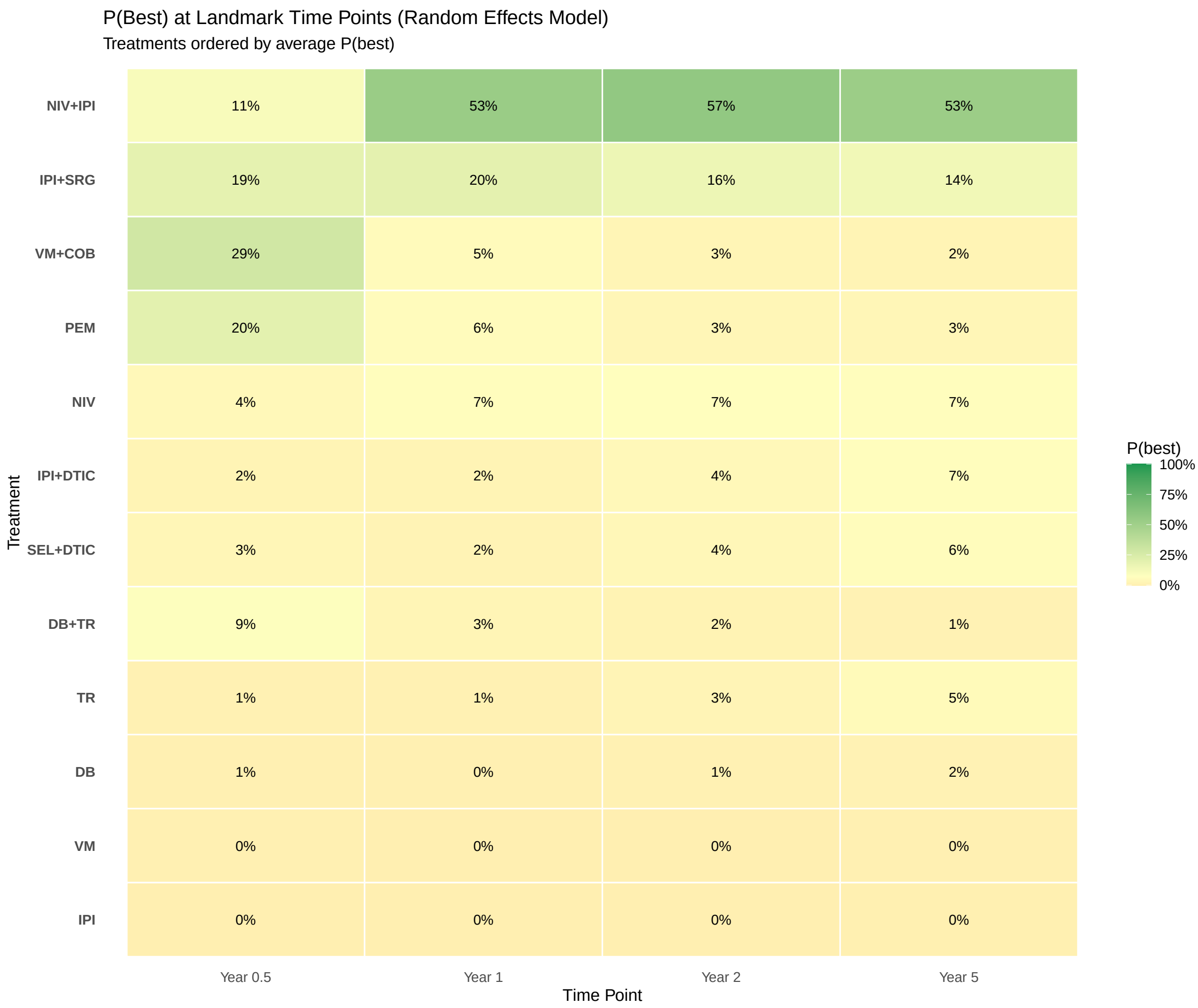

Fig. B.6 Melanoma network meta-analysis - heatmap of probability best at landmark time points from random effects network meta-analysis.
